# Role of contrast-enhanced ultrasound in evaluating the efficiency of ultrasound guided percutaneous microwave ablation in patients with renal cell carcinoma

**DOI:** 10.2478/raon-2013-0038

**Published:** 2013-10-08

**Authors:** Xin Li, Ping Liang, Jie Yu, Xiao-Ling Yu, Fang-Yi Liu, Zhi-Gang Cheng, Zhi-Yu Han

**Affiliations:** 1 Interventional Ultrasound Department of Chinese PLA General Hospital, Beijing, China; 2 Medical College of Nankai University, Tianjin, China

**Keywords:** contras enhanced ultrasound, microwave ablation, renal cell carcinoma

## Abstract

**Background:**

The aim of the study was to evaluate the efficiency and feasibility of contrast-enhanced ultrasound (CEUS) with Sonovue in assessing of renal cell carcinomas (RCCs) following ultrasound (US)-guided percutaneous microwave ablation (MWA).

**Patinets and methods:**

Seventy-nine patients (60 males and 19 females) with 83 lesions (mean size 3.2±1.6 cm) were treated by US-guided percutaneous MWA. The CEUS results of the third day after the ablation were compared with the synchronous contrast-enhanced computed tomography (CT)/magnetic resonance imaging (MRI) results and biopsy pathological results. The follow-up was performed by CEUS and CT/MRI after 1, 3, 6 months and every 6 months subsequently. The combination of clinical follow-up results and CT/MRI imaging findings was the reference standard of CEUS results for evaluating the therapeutic effect. The identification of residual or recurrence tumour was assessed by two blinded radiologists.

**Results:**

On the third day after MWA, CEUS showed 68 of 83 lesions (68/83, 81.9%) successfully ablated and 15 of 83 (18.1%) with residual tumours. Among residual tumours, 13 (86.7%) were confirmed by contrast-enhanced CT/MRI findings and biopsy results. The sensitivity, specificity, accuracy, positive and negative predictive value of CEUS evaluating the short-term MWA effectiveness were 100%, 97.1%, 97.6%, 86.7% and 100%, respectively. During the six years follow-up (median 26 months), the CEUS showed recurrence in 7 patients, and six of them achieved consistent results on CEUS and CT/MRI imaging. The sensitivity, specificity, accuracy, positive and negative predictive value for CEUS evaluating long-term MWA effectiveness were 85.7%, 98.7%, 97.6%, 85.7% and 98.7%, respectively.

**Conclusions:**

The post-procedural CEUS demonstrated as an effective and feasible method in evaluating a therapeutic effect of RCCs following MWA.

## Introduction

The incidence of renal cell carcinoma (RCC) has been increasing during the last decades of years. It accounts for 2.1% of all malignancies and is responsible for 1.5% of all cancer deaths worldwide.^1^ Thermal ablation including radiofrequency ablation (RFA) and cryoablation has rapidly expanded as an effective treatment option for patients who are sub optional candidates for surgical and laparoscopic renal surgery.^2^–^4^ Good local tumor control and survival rate have been recently reported for ultrasound (US)-guided percutaneous microwave ablation (MWA) for RCC, especially for small ones.^5^ Thermal ablation has rapidly expanded as an effective treatment option. To obtain a good therapeutic response, it is extremely important to assess the ablation completeness of the lesions as soon as possible after the treatment. Traditional computed tomography (CT) or magnetic resonance imaging (MRI) are often used to assess the ablation therapeutic efficacy.^6^ On the other hand the advantages of contrast-enhanced ultrasound (CEUS) as a safe, well tolerated, non-ionising imaging method with real-time multiplanar imaging, using nontoxic contrast agent have been well documented.^7^ CEUS has already been widely used in assessing thermal treatment in liver carcinoma with high a diagnostics accuracy.^8^ It has been proposed that CEUS was an alternative to CT and MRI in the follow-up of renal tumour treated with RFA.^9^ MWA results in higher thermal efficiency than RFA^10^, so for the rich blood organ (such as kidney) ablation, the tumour necrosis boundary may show a different CEUS pattern. To our knowledge, there is no paper reporting the application of CEUS in evaluating the therapeutic effect of MWA for RCC.

The aim of this study is to summarize our experience of the efficacy of CEUS in evaluating the short and long-term therapeutic effect in patients with RCC after MWA, using CT/MRI as reference standard. The ultimate goal is to find a simple, effective, convenient and safe evaluation technology to improve the ablation efficacy.

## Patients and methods

### Patients

This prospective study was approved by our institutional review board. The written informed consent for the procedure was obtained from each enrolled patient. Between April 2006 and June 2012, 84 RCC lesions in 80 patients were confirmed pathologically by US-guided percutaneous biopsy. Seventy-nine patients with 83 lesions were recruited and one patient with lesion diameter of 9.7 cm was excluded from the study due to the palliative treatment. All patients were treated on an inpatients basis. Patients’ characteristics were listed in Table 1. Pre- and post-ablation imaging (conventional US, CEUS and CT/MRI) were implemented to evaluate the characteristics of the tumours and ablation effectiveness. The tumour diagnosis was pathologically confirmed by intra-procedure biopsy.

### Ablation procedure

US-guided core needle biopsy (18 G, Bard, Japan) was performed just before the ablation during the same procedure. All ablations were performed using the microwave unit (KY-2000; Kangyou Medical, Nanjing, China) producing microwave energy (maximum power 100 W) through a 15-gauge cooled-shaft needle antenna with the emitting frequency of 2450 MHz. The patients underwent MWA under vein anesthesia, with a percutaneous US-guided and monitored approach using an Acuson Sequoia 512 scanner (Signature 10.2; Siemens Medical Solutions, Mountain View, Calif) with 3.5-5.0 MHz curved-array multi-frequency transducers. For tumours less than 2.0 cm, one antenna was inserted in the centre of the lesion, and for tumours measured 2.0 cm or larger, two antennae were used according to the characters of the lesion. The ablation time was from 300 to 600 seconds based on the tumour size. When the hyperechoic range covered the entire lesion, the microwave emitting was stopped.

### CEUS

The conventional US and CEUS were performed with an Acuson Sequoia 512 scanner and 4V1 transducer with frequencies of 1 – 4MHz. The CEUS imaging technique was contrast pulse sequencing (CPS) soft-ware which permitted real-time depiction of lesion blood perfusion under low mechanical index (MI < 0.2) in order to avoid microbubble disruption. A bolus injection of 1.0–1.2 ml of sulphur hexafluoride-filled microbubble contrast agent (Sonovue BR1; Bracco SpA; Milan, Italy) was administrated through a 20-gauge *canula* placed in the antecubital vein. The injection of Sonovue was followed by a flush of 5 ml 0.9% sodium chloride solution. All patients tolerated Sonovue application well. The entire examination was stored as a dynamic digital video file on the hard disk of the US scanner and recorded on a digital video recor der for further analysis.

### CT and MRI

Contrast-enhanced CT was performed in 47 patients with multi-detector row CT (Light speed 16; GE Medical Systems, Milwaukee, WI, USA) with a section thickness of 5 mm, a 1.35:1.0 pitch, 120 kV, and 250 mA, and contrast medium (iopromide, Ultravist 300; Schering, Berlin, Germany). Contrast-enhanced MRI was performed in 32 patients using a 1.5-T unit (Signa Echo-Speed; GE Medical Systems) with the sequences: spin-echo T1-weighted (500/15 [repetition time msec/echo time msec], 256 × 192 matrix, and two signals acquired); fat-suppressed T2-weighted respiratory-triggered fast spin-echo (3000–4000/102, 256 × 256 matrix, and three signals acquired); and fat-suppressed spin-echo T1-weighted (500/15, 256 × 192 matrix, and two signals acquired) sequence. F at-suppressed T1-weighted sequence was performed prior to and three times (scanning delay of 0 seconds for the corticomedullary phase, 70 seconds for the nephrographic phase, and 180 seconds for the excretory phase) after dynamic injection of 0.1 mmol of gadopentetate dimeglumine (Magnevist; Schering, Berlin, Germany) per kilogram of body weight. The subtraction of the postcontrast image from the precontrast image was performed for all time points.

### Follow-up

Three days after MWA, contrast-enhanced imagings (CEUS + CT, or CEUS + MRI) were performed to evaluate the ablation effect, then 1, 3, 6 months and every 6 months subsequently. T he CEUS results of the third day after the ablation were compared with the synchronous contrast-enhanced CT/MRI results. If CEUS and CT/MRI were both negative, the patient went into the follow-up stage. However, if the results were not consistent or both positive, then US-guided core needle biopsy was performed for the enhanced area and the patient underwent another ablation session for the possible residual area. The combination of clinical follow-up results and CT/MRI imaging results were reference standard of CEUS results for the long-term therapeutic effect. Every imaging was reviewed by two experienced radiologists (P.L., X.L.Y.).

### Imaging analysis

The criteria for CEUS imaging were: inflammatory congestion caused by MWA displayed uniformly circular enhancement around the necrosis zone. If irregular peripheral enhancement in scattered, nodular, or eccentric pattern was noted, this was thought to indicate the presence of residual, incompletely ablated tumor.^11^ This finding indicated the incomplete local treatment and a further ablation was considered if the patient still met the criteria for MWA. In patients with complete necrosis, a well-defined non-enhancing zone on CEUS and CT/MRI imaging was noted and the lesion shrank gradually over time.

### Statistical analysis

Statistics was performed by version SPSS 16.0 for windows statistical package (SPSS, Chicago, Ill). The baseline characteristic of patients and tumours were expressed as mean ±SD or median. Sensitivity, specificity, accuracy, positive predictive value (PPV) and negative predictive value (NPV) were calculated for CEUS in diagnosing accuracy of MWA effect for RCC.

## Results

Seventy-nine patients with 83 solid lesions treated with percutaneous MWA were enrolled in our study with mean age of 64.5±14.8 (22–82) years. There were 75 patients with one lesion and 4 patients with two. The lesions included clear cell carcinoma in 71 patients, papillary carcinoma in 6 patients, and chromophobe carcinoma in 2 patients. The mean size of the tumours was 3.2±1.6 cm. The sixty-eight of 83 (81.9%) lesions were successfully ablated in one session, 15 of 83 (18.1%) in two sessions. No severe procedure-related complications (including haematuria, pneumothorax, sepsis, renal infarction, skin burns, seeding and so on) were observed.

**FIGURE 1.**
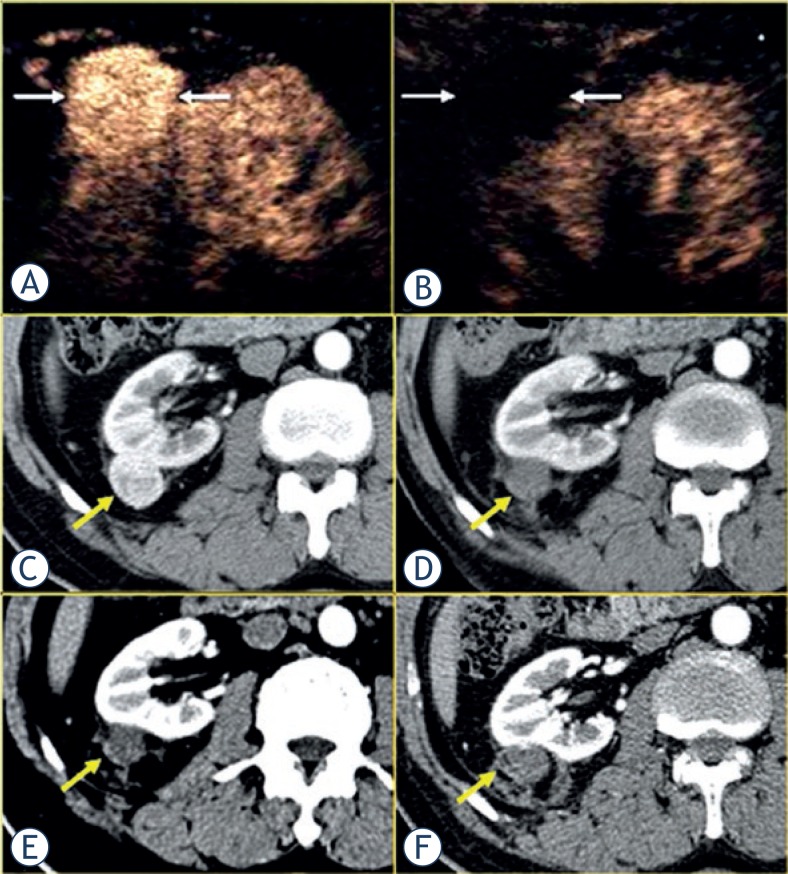
Images in 82-year-old female with a 3.9 cm × 3.7 cm renal clear cell carcinoma treated with microwave ablation (MWA). (A) Pre-ablation contrast-enhanced US scan showed one hyper-enhancement lesion exophyticly (arrow). (B) Seven days after ablation contrast-enhanced US showed the whole lesion no-enhancement continuously. (C) Transverse contrast-enhanced multidetector-row CT imaging showed one hyper-intense lesion pre-ablation and hypo-intense one in arterial phase 7 days (D), 1 month (E) and 6 months (F) after ablation.

**FIGURE 2.**
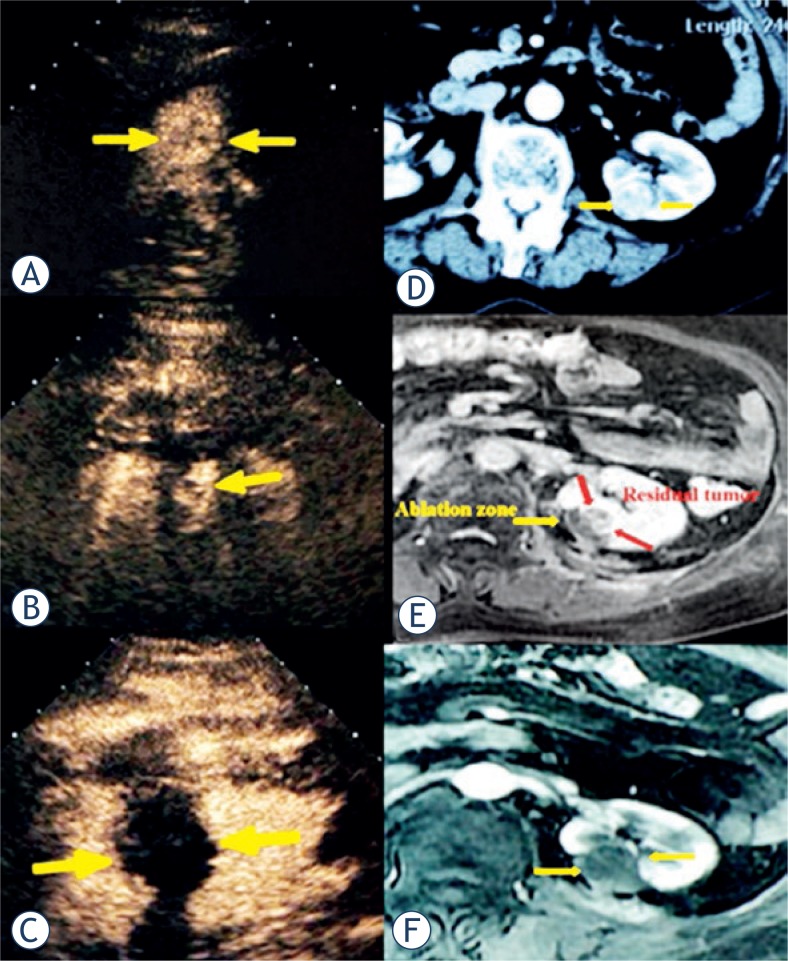
72-year-old female with a 4.5 cm × 3.9 cm renal clear cell carcinoma was treated with microwave ablation (MWA). (A) Pre-ablation contrast-enhanced US scan showed one hyper-enhancement lesion (arrow), (B) and 3 days after ablation showed a very small hyper-enhancement adjacent to the ablation zone. (C) Then the patient received another ablation and reached completed necrosis. Transverse contrast-enhanced MR imaging showed one hyper-intense lesion a little exophytic and adjacent to renal pelvis in arterial phase pre-ablation and showed the same hyper-intense (residual tumour) (red arrow) homochronously (D), then no-intense after another ablation (F).

On the third day after MWA, CEUS showed that 68 of 83 lesions (82.9%) were successfully ablated. The results of CEUS were confirmed by contrast-enhanced CT/MRI three days after MWA (Figure 1). Fifteen of 83 (18.3%) lesions appeared residual tumour on CEUS. Among them biopsy results verified 13 residual tumours (Figure 2) and the second CEUS-guided ablations were done. The sensitivity, specificity, accuracy, PPV and NPV of CEUS evaluating instant effect of MWA of RCC were 100%, 97.1%, 97.6%, 86.7% and 100%, respectively (Table 2).

**FIGURE 3.**
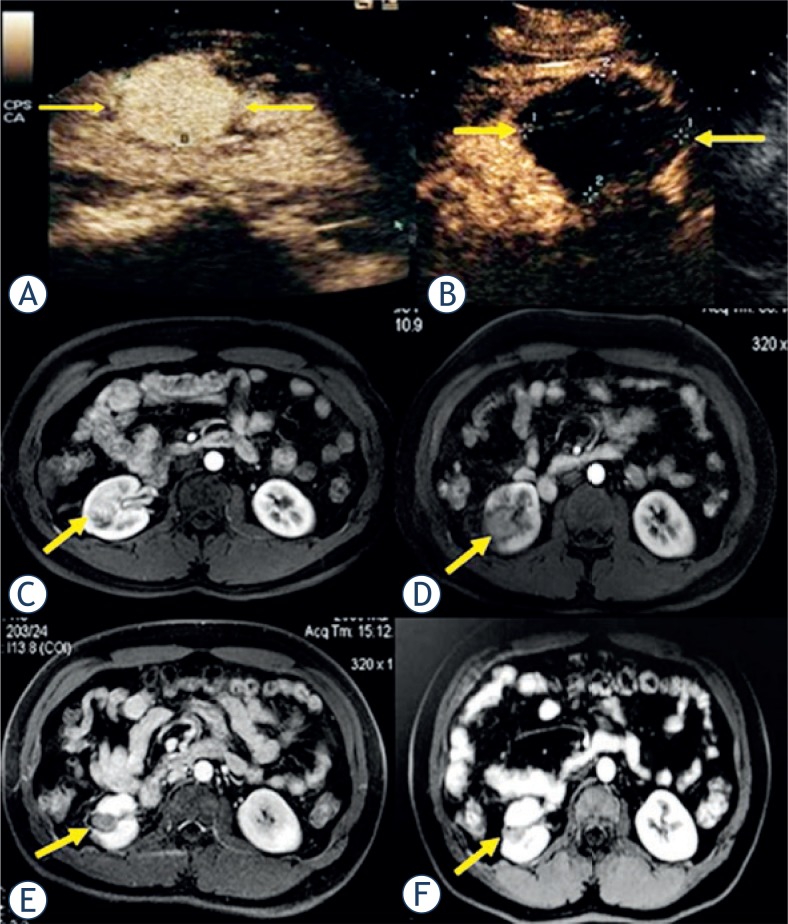
Pre-ablation contrast-enhanced US showed a hyper-enhancement 2.4 cm × 1.9 cm renal clear cell carcinoma in renal parenchyma in a 45-year-old man (A, arrow). Transverse contrast-enhanced MR imaging showed one hyper-intense lesion adjacent to renal pelvis pre-ablation (C) and hypo-intense in arterial phase 7 days (D), 1 year (E) and 2 years (F) after ablation, and the lesion shrunk gradually obviously.

**FIGURE 4.**
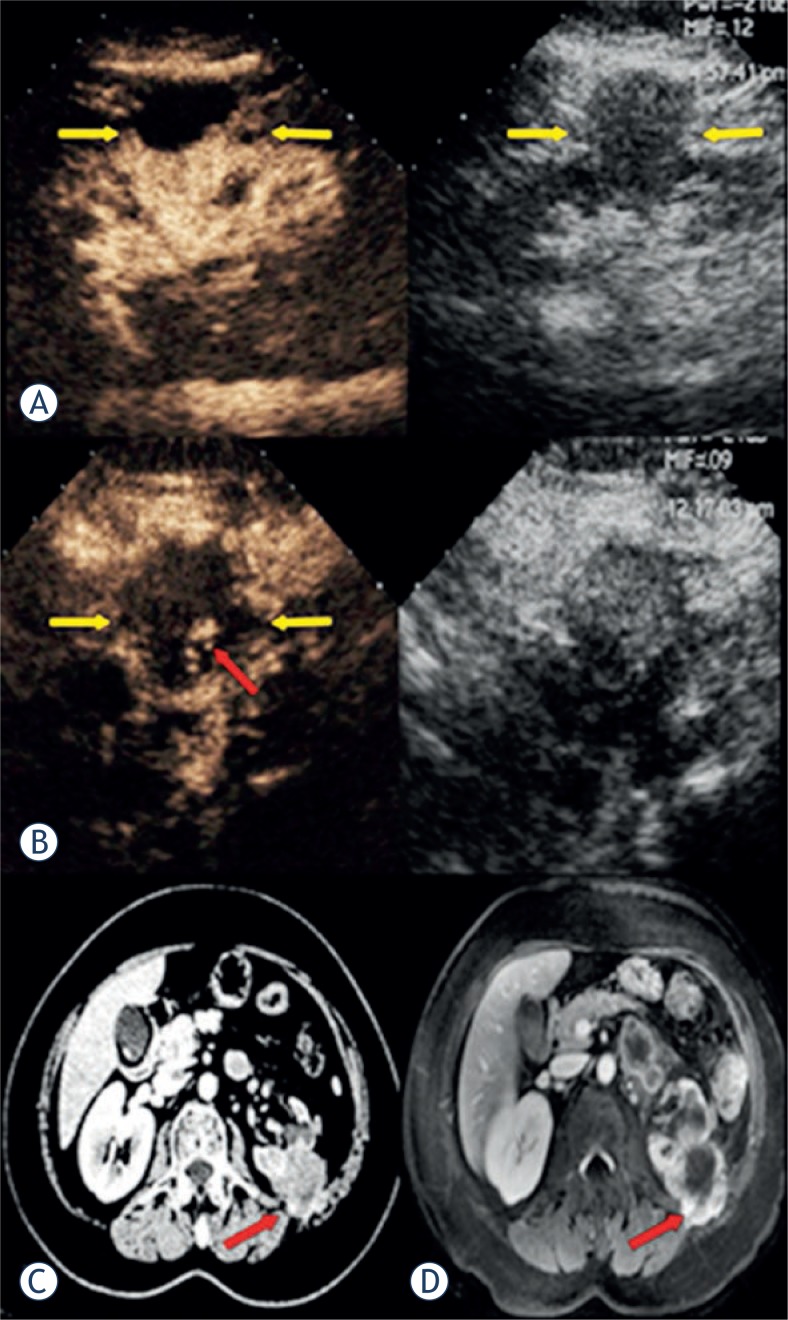
Pre-ablation contrast-enhanced US showed a 4.7 cm × 3.9 cm exophyticly renal papillary cell carcinoma heterogeneous hyper-enhancement with no-enhancement zone in a 56-year-old woman (A, arrow), and 1 month after ablation contrast-enhancement US showed an hyper-enhancement area in cortical phase which was diagnosed as abnormal perfusion (B, red arrow), while the corresponding period CT and MRI showed the hyper-enhancement in article phase and diagnosed as a recurrence tumour (C, D, red arrow). Then the patient received biopsy and another ablation and verified the recurrence.

During the median follow-up period of 26 months (rang 3–74 months), seventy-six patients survived and three patients died of heart failure, gastrorrhagia and multiple organs failure at 37, 39, 61 months after the ablation respectively. Seventy-six (76/83) lesions showed a completed ablation during the follow-up (Figure 3). Seven (7/83) lesions were detected recurrent tumour, confirmed by CEUS and CT/MRI in six patients with consistency. One patient with the lesion (max diameter 4.7 cm, located exophytic and adjacent to intestinal tract) showed a completed ablation at 6 months after the ablation on CEUS, but local tumour progression was detected on MRI and then confirmed by subsequent nephrectomy (Figure 4). At 15 months after the ablation CEUS detected recurrence in one patient with max diameter 5.8 cm lesion located endophytic close to pelvis but the result was denied by MRI, while the CT imaging and biopsy result verified the result of CEUS and the CT and CEUS confirmed complete necrosis during 74 months follow-up. The sensitivity, specificity, accuracy, PPV and NPV for CEUS detecting residual or recurrence tumour during the follow-up period were 85.7%, 98.7%, 96.7 %, 85.7% and 98.7%, respectively (Table 3).

## Discussion

The image-guided thermal ablation has been widely used for renal lesions treatment on account of the technology promotion, and obtained a favourable curative effect parallel to nephron-sparing surgery especially for small ones.^4^,^12^ To achieve radical clinical results after MWA of renal tumours, it is important to select suitable patients. Indications for MWA of RCC including patients with small lesions (size less than 4 cm), patients of advanced age or poor surgical candidates due to significant comorbidities, those with single kidney or multiple tumours in both kidneys; and patients with ablation preference. In addition, the timely and accurate evaluation of the therapeutic effects is also momentous for promoting the ablation effect. The CT/MRI as traditional imaging in evaluating the ablation treatment efficacy has some limitations. CEUS with the second generation contrast agent Sonovue as a useful, convenient, no-hepatoctoxicity and no-nephrotoxicity tool has been widely used and provided abundant diagnosis and assessment information, especially suitable for renal function impaired patients. There were several reports on CUES assessment of RFA or cryoablation of renal lesions^13^–^15^, but in the MWA field, a study on CEUS evaluation was seldom.

To investigate the role of CEUS in evaluating the MWA of kidney, we performed the short-term (three days) and long-term observation by using CEUS as evaluation tool after MWA of RCC with promising results.

Though CEUS confirmed comparable results with CT/MRI in evaluation of RCC ablation effect, CEUS had the advantages in real-time showing the ablation zone, the surrounding renal parenchyma and the renal vessels that may provide potential superiority for detecting local tumour progression. However, just as conventional US, CEUS is subject to unclear lesion display for abdominal gas shielding or relative operator dependence of US. The optimal time of CEUS to evaluate the therapeutic effects after thermal ablation was another important point to promote the diagnosis ability. The timely and accurate detection of the residual tumour was important for choosing a proper treatment as early as possible to improve the tumour-free survival rate. One week to one month interval after the ablation were preferred by some researchers.^13^–^16^. In our study the initial evaluation time was chosen as the third day after MWA due to the fact that congestion zone 72 hours after the thermal ablation was less evident than within 24 hours after the ablation, which may decrease the distraction for the CEUS and another session could be provided timely if necessary.

This study has some limitations as well. Firstly, post-ablation biopsy with pathological results was not performed in all lesions due to a bleeding risk. Some authors had pointed out that the use of needle biopsy had some limitation in depicting residual lesions after thermal ablation.^17^ Secondly, to some extent, the CEUS imaging evaluation was depended on the experience of the radiologists. Larger series and longer follow-up period are needed to further confirm the results of our study. Also comparative investigation of CEUS evaluation in MWA and RFA of RCC is mandatory.

## Conclusions

Ultimately the post-procedural CEUS appearances to be a convenient, repeatable, less-toxically technology with high diagnosis accuracy and plays a promising role in evaluating the therapeutic effect of RCCs following MWA.

## Figures and Tables

**TABLE 1.** Patients and renal cell carcinomas characteristics

**Characteristics**	**Data**

**Patients**	
Male/Female	61/19

Age (mean±sd [rang]) years	64.5±14.8 (22–82)
Bilateral tumours	4 (4.8%)

**Renal cell carcinomas**	

Size (cm)	
mean±sd [rang]	3.2±1.6 (0.6–7.8)
≤4	66 (78.6%)
>4	18 (21.4%)
Location(side)	
Right/Left	48/36
Location(type)	
Exophytic^*^	16 (19.0%)
Intraparenchymal^**^	46 (54.8%)
Endophytic ^***^	22 (26.2%)
Histological diagnoses by biopsy	
Renal clear cell carcinoma	76 (90.5%)
Renal papillary cell carcinoma	6 (7.1%)
Chromophobe renal cell carcinoma	2 (2.4%)
Median follow-up (month)	26 (3–74)

* Exophytic lesions were defined as nodules beyond the renal contour with no component extending into the renal sinus.

** Intraarenchymal lesions were defined as nodules confined within the parenchyma without contour bulging or sinus extension.

*** Endophytic lesions were defined as nodules that extended into the renal sinus and were in close proximity to the collecting structures or the ureters.

**TABLE 2.** Evaluation performance of contrast-enhanced ultrasound (CEUS) on assessment of the therapeutic effects for renal cell carcinomas on the third day after microwave ablation

**Follow-u CEUS**	**Reference standard**	
	**Residual**	**No residual**
Residual	13	2
No residual	0	68

**TABLE 3.** Evaluation performance of contrast-enhanced ultrasound (CEUS) on assessment of the therapeutic effects after microwave ablation for renal cell carcinomas during the follow-up

**Follow-u CEUS**	**Reference standard**	
	**Recurrence**	**No recurrence**
Recurrence	6	1
No recurrence	1	75
